# Supplementary material for: Non-nucleoside reverse transcriptase inhibitor-based combination antiretroviral therapy is associated with lower cell-associated HIV RNA and DNA levels compared to protease inhibitor-based therapy
Source: eLife. 2021 Aug 13;10:e68174. doi: 10.7554/eLife.68174 (PMC8460250; doi:10.7554/eLife.68174)
Supplement: Supplementary file 1. — (a) Variables associated with cell-associated HIV US RNA and total HIV DNA levels in the COmorBidity in Relation to AIDS (COBRA) cohort. (b) Variables associated with cell-associated HIV US RNA and total HIV DNA levels in the Adherence Improving Self-Management Strategy (AIMS) cohort. [file elife-68174-supp1.docx]

**Supplementary file 1a.** Variables associated with cell-associated HIV unspliced (US) RNA and total HIV DNA levels in the COBRA cohort.

| **Dependent variable** | **Explanatory variable** | **Category** | **Univariable analyses** | | **Multivariable analysis** | |
| --- | --- | --- | --- | --- | --- | --- |
|  |  |  | **B (95% CI)** | ***P*** | **B (95% CI)** | ***P*** |
| US RNA, log_10_ copies/μg RNA | Duration of continuous virological suppression, per year | - | -0.01 (-0.05-0.04) | 0.81 |  |  |
|  | Plasma HIV RNA zenith, per log_10_ copies/mL | - | 0.37 (0.06-0.69) | 0.02 | 0.37 (0.07-0.68) | 0.02 |
|  | Current CD4+ count, per 100 cells/mm^3^ | - | -0.01 (-0.08-0.05) | 0.66 |  |  |
|  | CD4+ count nadir, per 100 cells/mm^3^ | - | -0.05 (-0.16-0.06) | 0.40 |  |  |
|  | Age, per 10 years | - | 0.00 (-0.01-0.02) | 0.72 |  |  |
|  | ART regimen | PI | 0.29 (0.04-0.54) | 0.02 | 0.30 (0.04-0.55) | 0.02 |
|  |  | NNRTI | 1 | - |  | - |
|  | NRTI backbone | 3TC+TDF | 0.05 (-0.45-0.54) | 0.85 |  |  |
|  |  | ABC+3TC | 0.40 (-0.16-0.95) | 0.16 |  |  |
|  |  | FTC+TDF | 1 | - |  |  |
| Total DNA, log_10_ copies/million PBMC | Duration of continuous virological suppression, per year | - | -0.01 (-0.03-0.01) | 0.52 |  |  |
|  | Plasma HIV RNA zenith, per log_10_ copies/mL | - | 0.37 (0.18-0.56) | 0.0002 | 0.38 (0.19-0.56) | 0.0001 |
|  | Current CD4+ count, per 100 cells/mm^3^ | - | -0.02 (-0.14-0.11) | 0.82 |  |  |
|  | CD4+ count nadir, per 100 cells/mm^3^ | - | -0.04 (-0.12-0.04) | 0.35 |  |  |
|  | Age, per 10 years | - | -0.06 (-0.43-0.31) | 0.75 |  |  |
|  | ART regimen | PI | 0.25 (-0.01-0.50) | 0.06 | 0.25 (0.00-0.50) | 0.048 |
|  |  | NNRTI | 1 | - |  | - |
|  | NRTI backbone | 3TC+TDF | -0.04 (-0.32-0.24) | 0.79 |  |  |
|  |  | ABC+3TC | 0.17 (-0.68-1.03) | 0.69 |  |  |
|  |  | FTC+TDF | 1 | - |  |  |

**Supplementary file 1b.** Variables associated with cell-associated HIV unspliced (US) RNA and total HIV DNA levels in the AIMS cohort.

| **Dependent variable** | **Explanatory variable** | **Category** | **Univariable analyses** | | **Multivariable analysis** | |
| --- | --- | --- | --- | --- | --- | --- |
|  |  |  | **B (95% CI)** | ***P*** | **B (95% CI)** | ***P*** |
| US RNA, log_10_ copies/μg RNA | Duration of continuous virological suppression, per year | - | -0.06 (-0.10- -0.03) | 0.0003 | -0.04 (-0.08-0.00) | 0.04 |
|  | Plasma HIV RNA zenith, per log_10_ copies/mL | - | 0.13 (0.00-0.27) | 0.05 | 0.03 (-0.08-0.15) | 0.59 |
|  | Current plasma HIV RNA | Detectable | 0.15 (-0.09-0.40) | 0.22 |  |  |
|  |  | Undetectable | 1 | - |  |  |
|  | Current CD4+ count, per 100 cells/mm^3^ | - | -0.05 (-0.10- -0.01) | 0.02 | -0.04 (-0.09-0.01) | 0.13 |
|  | CD4+ count nadir, per 100 cells/mm^3^ | - | -0.06 (-0.14-0.01) | 0.11 |  |  |
|  | Age, per 10 years | - | 0.01 (-0.07-0.09) | 0.75 |  |  |
|  | Gender | Male | 0.02 (-0.34-0.37) | 0.92 |  |  |
|  |  | Female | 1 | - |  |  |
|  | ART regimen | PI | 0.29 (0.09-0.50) | 0.006 | 0.24 (0.04-0.45) | 0.02 |
|  |  | NNRTI | 1 | - |  | - |
|  | NRTI backbone | FTC+TDF | 0.18 (-0.13-0.48) | 0.25 |  |  |
|  |  | AZT+3TC | -0.06 (-0.28-0.16) | 0.59 |  |  |
|  |  | 3TC+TDF | 1 | - |  |  |
|  | Adherence to ART, per 10% | - | -0.03 (-0.07-0.01) | 0.20 |  |  |
| Total DNA, log_10_ copies/million PBMC | Duration of continuous virological suppression, per year | - | -0.04 (-0.08- -0.01) | 0.01 | -0.04 (-0.07-0.00) | 0.03 |
|  | Plasma HIV RNA zenith, per log_10_ copies/mL | - | 0.01 (-0.11-0.12) | 0.92 |  |  |
|  | Current plasma HIV RNA | Detectable | 0.30 (-0.09-0.69) | 0.13 |  |  |
|  |  | Undetectable | 1 | - |  |  |
|  | Current CD4+ count, per 100 cells/mm^3^ | - | -0.02 (-0.10-0.05) | 0.57 |  |  |
|  | CD4+ count nadir, per 100 cells/mm^3^ | - | -0.04 (-0.36-0.27) | 0.78 |  |  |
|  | Age, per 10 years | - | 0.05 (-0.05-0.16) | 0.29 |  |  |
|  | Gender | Male | 0.07 (-0.12-0.25) | 0.47 |  |  |
|  |  | Female | 1 | - |  |  |
|  | ART regimen | PI | 0.30 (0.02-0.58) | 0.03 | 0.26 (-0.05-0.56) | 0.10 |
|  |  | NNRTI | 1 | - |  |  |
|  | NRTI backbone | FTC+TDF | -0.16 (-0.45-0.14) | 0.30 |  |  |
|  |  | AZT+3TC | -0.17 (-0.40-0.06) | 0.15 |  |  |
|  |  | 3TC+TDF | 1 | - |  |  |
|  | Adherence to ART, per 10% | - | -0.01 (-0.04-0.02) | 0.46 |  |  |
